# Entry and Fusion of Emerging Paramyxoviruses

**DOI:** 10.1371/journal.ppat.1000881

**Published:** 2010-06-24

**Authors:** Rebecca Ellis Dutch

**Affiliations:** Department of Molecular and Cellular Biochemistry, University of Kentucky, Lexington, Kentucky, United States of America; University of California San Francisco, United States of America

Paramyxoviruses are a family of non-segmented RNA viruses that includes major human pathogens such as measles virus and respiratory syncytial virus (RSV) and significant animal viruses like rinderpest [1]. In recent years, several new paramyxoviruses have been identified, further increasing the breadth and importance of this viral family. While many elements of the fusion and entry mechanisms of these recently identified pathogens are conserved, there are interesting differences, including variations in receptor binding, cell tropism, fusion (F) protein proteolytic activation, and triggering of membrane fusion. Thus, study of their entry mechanisms has highlighted the diversity of these critical events in the family.

## Paramyxoviruses: An Expanding Group of Important Viral Pathogens

**Figure 1:**
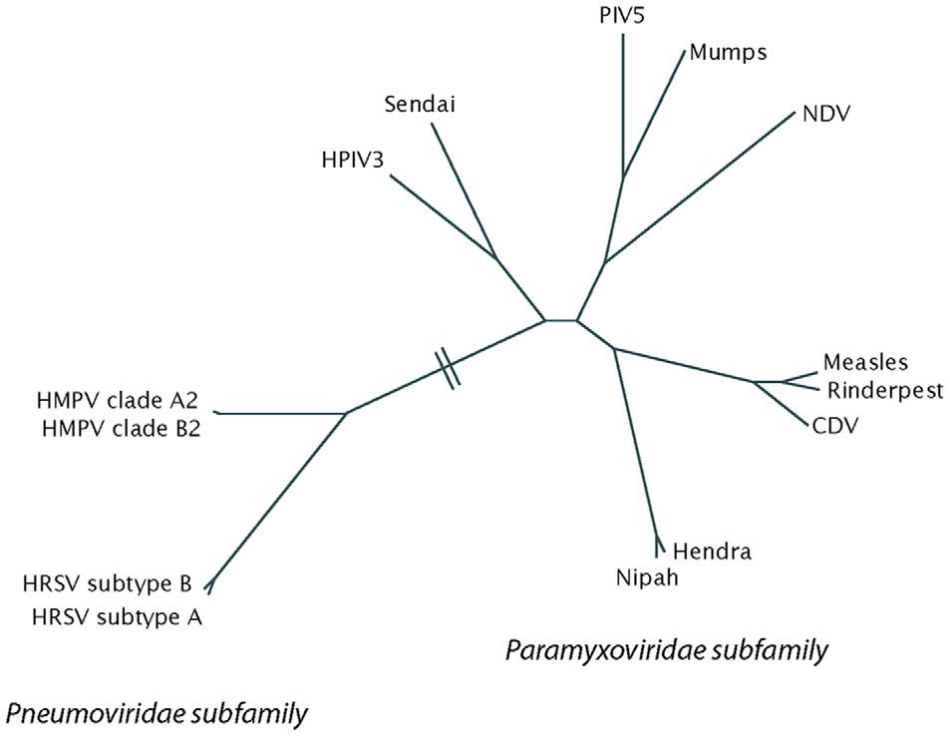
A phylogenetic tree of the paramyxovirus family, built using fusion protein sequence comparison. The tree was generated from a PROMALS multiple sequence alignment using PROTDIST and FITCH from the PHYLIP 3.65 software package and displayed using HYPERTREE 1.0.0. CDV, canine distemper virus; HMPV, human metapneumovirus; HPIV3, human parainfluenza virus 3; HRSV, human respiratory syncytial virus; NDV, Newcastle disease virus; PIV5, parainfluenza virus 5.

Hendra virus and Nipah virus are the only identified zoonotic members of the paramyxovirus family, and both are highly pathogenic in humans [2]. Hendra virus infection has resulted in multiple horse and four human fatalities since its emergence in Australia in 1994, with outbreaks in 2008 and 2009 leading to rising concern in the Australian horse breeding industry. Nipah virus emerged in Malaysia in 1999, causing an outbreak of viral encephalitis that led to 105 human fatalities out of 265 reported cases. Containment of the 1999 Nipah virus epidemic required the sacrifice of more than 1 million swine. Continued Nipah outbreaks have occurred in Southeast Asia, with mortality rates of up to 70% and suspected human-to-human transmission. Numerous molecular features have led to the placement of Hendra and Nipah viruses within a new genus in the paramyxovirus family, the henipaviruses (Figure 1). The principal reservoir species for both viruses is thought to be *Pteropus* fruit bats, but a number of other species have been shown to be susceptible to infection [3].

Human metapneumovirus (HMPV) was first identified in 2001, but unlike Hendra and Nipah, HMPV is not a new human virus resulting from zoonotic transmission. Instead, HMPV is a long-term human pathogen that was only identified by careful analysis of samples from children with respiratory tract disease for which an etiological agent had not been identified [4]. Subsequent studies indicate that HMPV is a major causative agent of respiratory tract infections worldwide, causing between 5% and 15% of lower respiratory tract infections in young children [5]. HMPV has been circulating in the human population since at least 1958 [4]. Sequence analysis places HMPV in the Pneumovirinae subfamily, along with RSV.

## Fusion Mechanisms: Conserved Features in Newly Identified Paramyxoviruses

To enter host cells, paramyxoviruses must go through the key steps of viral attachment to the target cell, followed by fusion of the viral membrane to a host cell membrane [6]. Two major viral glycoproteins promote these events: the attachment protein facilitates primary receptor binding of the virus to the target cell, while the F protein promotes the subsequent membrane fusion events. Both events are hypothesized to occur at the cell surface in a neutral pH environment. Interactions between the F protein and the homotypic attachment protein are hypothesized to control initiation of the fusion process for most paramyxoviruses, though the mechanistic details of triggering control remain elusive. Once begun, fusion is promoted by a series of conformational changes in the F protein that first lead to insertion of a hydrophobic region (termed the fusion peptide) into the target membrane, forming a protein bridge between the two membranes. Additional conformational changes lead to formation of a helical bundle, formed by interactions between two heptad repeat regions that do not interact in the prefusion form of the protein [1], and subsequent membrane fusion.

A number of factors point to an overall conserved mechanism of fusion promotion among the paramyxovirus F proteins. While there is considerable heterogeneity at the amino acid level, F proteins from both established and newly identified paramyxoviruses display conserved positioning of cysteine, glycine, and proline residues, suggesting an overall conservation of structure. F proteins also contain similarly placed fusion peptide and heptad repeat regions. Peptides corresponding to the F protein heptad repeat regions have been shown to block fusion and entry for previously studied paramyxoviruses, and similar peptides inhibit Hendra, Nipah, and HMPV fusion and entry, indicating that the requirement for formation of the final helical bundle is a conserved feature [2], [6]. Like previously identified members of the family, fusion activity of the Hendra and Nipah F proteins requires the presence of a viral attachment protein, though either the Hendra or Nipah attachment protein can be used interchangeably [6]. As was seen with measles virus, recent evidence suggests that fusion activity for the Hendra and Nipah F proteins is inversely proportional to the strength of the F attachment protein interactions, in contrast to results from other paramyxovirus systems such as Newcastle disease viruses [7], suggesting slightly different mechanisms of control of fusion initiation.

## Identification of Ephrin B2 as the Receptor for Hendra and Nipah Viruses: Implications for Tissue and Species Range

Initial characterization of the activity of the Hendra and Nipah attachment proteins indicated interaction with a protein receptor, as is the case for measles virus attachment protein, rather than the sialic acid binding observed for most paramyxovirus attachment proteins [3]. Further study identified EphrinB2 as the receptor for the Hendra and Nipah viruses [8], [9], with EphrinB3 later shown to serve as an additional receptor for both viruses. Structural analysis of Nipah G alone or in complex with Ephrin B3 interestingly showed little conformational change upon receptor binding, suggesting that only subtle alterations in the attachment protein lead to F protein activation [10]. EphrinB2 and B3 serve as ligands for the Eph tyrosine receptor family, and their cellular expression in neurons, arterial endothelial cells, and smooth muscle is consistent with the tissue distribution observed during Hendra and Nipah infection [3]. EphrinB2 and B3 are also highly conserved between species, fitting with the large number of species shown to be infected by these pathogens. EphrinB2 and B3 from multiple infectable species, including human, horse, pig, cat, dog, and bat, have been shown to serve as functional receptors for Hendra and Nipah [11], suggesting that the conserved expression of this receptor plays an important role in the unusually broad host range of these pathogens. Interestingly, murine EphrinB2 can serve as a functional receptor for these viruses, but mice are resistant to henipavirus infection, indicating that additional factors modulate overall host range.

## Cathepsin L Processing of the Hendra and Nipah F Proteins: A New Paradigm for Fusion Protein Proteolytic Activation

**Figure 2:**
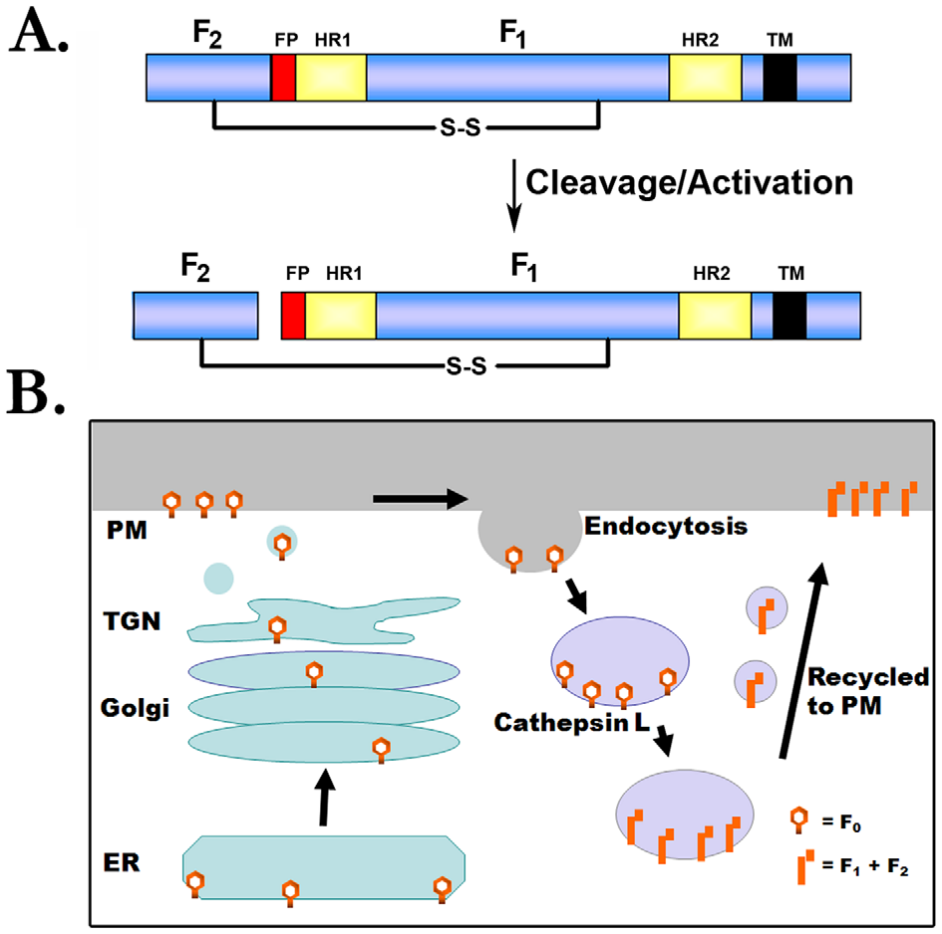
Proteolytic processing of the henipavirus fusion proteins. (A) Schematic of F protein cleavage/activation. FP, fusion peptide; HR1 and HR2, heptad repeat; S-S, disulfide bond; TM, transmembrane domain. (B) Model for proteolytic processing of the henipavirus F proteins. The Hendra or Nipah F proteins are synthesized in the endoplasmic reticulum (ER), transit through the secretory pathway to the plasma membrane (PM), and are subsequently endocytosed. Following interaction with cathepsin L at an undetermined point in the endocytic pathway, the cleaved protein is recycled to the plasma membrane.

Like other paramyxovirus F proteins, the Hendra and Nipah virus F proteins are initially synthesized as a precursor (F_0_) that must be proteolytically processed to two subunits (F_1_ and F_2_) to be fusogenically active (Figure 2A). For the majority of F proteins, this critical proteolytic processing event is promoted by furin, a cellular protease present primarily in the *trans*-Golgi network. Interestingly, the mechanism for proteolytic activation of the henipavirus F proteins is completely novel. Furin is clearly not involved, as there is no furin consensus at the cleavage site, furin inhibitors have no effect on henipavirus F processing, and processing occurs efficiently in furin-negative cell lines [2]. Instead, inhibitors or shRNA knock-downs of the cellular endosomal protease cathepsin L were shown to inhibit cleavage of the Hendra and Nipah F proteins, and in vitro studies confirmed proteolytic cleavage of the henipavirus F proteins at a single specific site by purified cathepsin L [6], [12]. To facilitate this key interaction with cathepsin L, endocytosis of the Hendra F protein [13] and the Nipah F protein [14] must occur, followed by a retrafficking event to the cell surface after proteolytic processing (Figure 2B). As cleaved F protein is present within the packaged virion [3], this complex trafficking of the henipavirus F proteins through the endosomal pathway occurs prior to viral assembly. Interestingly, the Hendra G attachment protein does not follow this complicated trafficking pathway, indicating that the critical attachment protein: fusion protein interactions needed for fusion occur only after F protein endocytic trafficking and proteolytic cleavage [15]. The reason for this novel activation pathway is unclear, though it is intriguing to note that Ebola virus and SARS coronavirus also have a role for cathepsin L at some point during the viral life cycle, and like Hendra and Nipah virus, the reservoir species for these important pathogens is thought to be bats. Future studies on protease profiles in bat cells may shed light on the reason for the unusual role of cathepsins in the life cycles of these pathogens.

## Analysis of HMPV: Novel Findings on Attachment, Fusion, and the Entry Pathway

While analysis of henipavirus entry mechanisms has broadened diversity related to receptor usage and proteolytic activation, study of HMPV entry has further illuminated differences between the Paramyxovirinae and Pneumovirinae sub-families (Figure 1). Specific attachment protein∶fusion protein interactions are needed for fusion promoted by Paramyxovirinae glycoproteins, with the exception of the parainfluenza virus 5 F protein, which can promote fusion in the absence of its attachment protein, though at a significantly decreased level [1]. In contrast, a recombinant RSV lacking the attachment protein can replicate in some types of cultured cells [5], indicating a significantly decreased requirement for this protein during attachment and entry. The altered role of the attachment protein is even more striking in HMPV, as recombinants lacking the attachment protein are competent for replication in African green monkeys, though there is decreased replication in the lower respiratory tract [16]. Studies of cell–cell fusion found that the HMPV F protein by itself was capable of promoting both binding and fusion, and no stimulation by the attachment protein was observed [17]. Combined with the viral studies, these results suggest that HMPV F can interact with a receptor(s) on the target cells, though the identity of the F receptor remains to be defined. These findings also raise the important question of what triggers the HMPV F protein to initiate fusion, as in this case fusion initiation is clearly not controlled by interactions with the attachment protein. Interestingly, recent studies indicate that low pH can serve as a fusion trigger [17] for at least a portion of the HMPV strains [18], and specific residues that could promote low-pH-induced conformational change through an electrostatic repulsion mechanism have been identified [19]. In addition, endocytosis has very recently been implicated in both HMPV [19] and RSV [20] entry, indicating that the virus will come in contact with the acidic pH of the endosomes during entry.

Overall, the study of entry and membrane fusion of recently identified paramyxoviruses has broadened the paradigms of receptor usage, F protein proteolytic activation, and membrane fusion triggering. Future work will continue to define how these variations modulate infectivity and pathogenicity in this important viral family.
